# Temsirolimus, an mTOR inhibitor, enhances anti-tumour effects of heat shock protein cancer vaccines

**DOI:** 10.1038/bjc.2011.15

**Published:** 2011-02-01

**Authors:** Y Wang, X-Y Wang, J R Subjeck, P A Shrikant, H L Kim

**Affiliations:** 1 Division of Urology, Department of Surgery, Cedars-Sinai Medical Center, 8635 West 3rd Street, Suite 1070W, Los Angeles, CA 90048, USA; 2 Department of Human and Molecular Genetics, Virginia Commonwealth University, Richmond, VA, USA; 3 Department of Cell Stress Biology, Roswell Park Cancer Institute, Buffalo, NY, USA; 4 Department of Immunology, Roswell Park Cancer Institute, Elm and Carlton Streets, Buffalo, NY 14263, USA

**Keywords:** temsirolimus, mTOR, melanoma, renal cell carcinoma, tumour vaccine, heat shock protein

## Abstract

**Background:**

Temsirolimus is a mammalian target of rapamycin (mTOR) inhibitor and rapamycin analogue that is approved for treating advanced renal cell carcinoma (RCC). It is being actively evaluated in clinical trials for melanoma. The mTOR inhibitors are also immunosuppressants and are used clinically to prevent rejection following solid-organ transplant. Novel immunotherapies are being actively developed for immunoresponsive tumours, such as RCC and melanoma.

**Methods:**

Immune-modulating effects of temsirolimus were characterised when used in combination with cancer vaccines targeting RCC (RENCA) and melanoma (B16). Cancer vaccines were recombinant tumour-specific proteins (CA9 or gp100), and recombinant heat shock protein (HSP; hsp110) served as the immune adjuvant.

**Results:**

In murine models, temsirolimus enhanced the anti-tumour activity of cancer vaccines used to treat established RENCA and B16 tumours. A tumour prevention model established that the enhanced anti-tumour activity associated with temsirolimus was immune mediated. In mice treated with an HSP-based anti-tumour vaccine, temsirolimus-treated CD8 T cells had greater interferon-*γ* and cytotoxic T-cell responses when compared with mice treated with vaccine alone. Temsirolimus also enhanced the formation of CD8 memory cells following administration of HSP-based cancer vaccine.

**Conclusion:**

These results provide a rationale for combining mTOR inhibitor with immunotherapy when treating immunoresponsive tumours.

The mammalian target of rapamycin (mTOR) is a pivotal regulator of cell proliferation. (Thomson *et al*, 2009) The mTOR protein integrates diverse signals originating from growth factors, energy status, and cellular stress, and promotes mRNA translation and cell proliferation. Therefore, inhibition of mTOR function has broad anti-proliferative effects. Temsirolimus and evirolimus are mTOR inhibitors that are currently approved for treatment of advanced renal cell carcinoma (RCC), and both agents are being actively investigated in clinical trials for a large number of malignancies. The rationale for using mTOR inhibitors to treat malignancies has focused on the direct, growth-inhibitory effects of mTOR inhibitors.

However, mTOR inhibitors are expected to have important immune-modulating effects. Rapamycin is the prototypic mTOR inhibitor, and is widely used to suppress the immune system and prevent rejection of solid-organ transplants. For this reason, recent reports attributing immune-stimulating effects to mTOR inhibition are surprising. These reports directly show that rapamycin can enhance vaccines targeting bacteria (Jagannath *et al*, 2009) or virus (Araki *et al*, 2009) in mouse models. Therefore, it may be possible to use mTOR inhibitors to enhance vaccines targeting cancers. Understanding the immune effects of mTOR inhibition is particularly relevant for immunoresponsive diseases, such as RCC and melanoma, for which novel cancer vaccines are being actively developed. The mTOR inhibitors are approved for advanced RCC patients; therefore, the possibility of combing a new cancer vaccine with an established therapy that has unexpected immunostimulatory properties is attractive.

We investigated the effects of temsirolimus when used in combination with heat shock protein (HSP)-based anti-tumour vaccines in experimental tumour models for RCC and melanoma. The HSPs are immune adjuvants that are capable of binding tumour antigens and interacting with antigen-presenting cells to mediate a CD8 T-cell response (Udono *et al*, 1994; Todryk *et al*, 2000; Castelli *et al*, 2001; Matsutake and Srivastava, 2001). In our survey of the immune effects mediated by the combination of cancer vaccine and mTOR inhibition, temsirolimus had both immunosuppressive and immunostimulatory properties. Temsirolimus decreased the proliferation of activated T cells, increased the relative abundance of regulatory T cells, and suppressed dendritic cell (DC) function. However, temsirolimus enhanced the activation of effector CD8 T cells and development of memory CD8 T cells. The net effect of temsirolimus was to enhance the anti-tumour response to HSP-based tumour vaccines.

## Materials and Methods

### Mice and cell lines

Wild-type C57BL/6 and BALB/C mice, 6–8 weeks old, were purchased from NCI (Frederick, MD, USA) and housed under pathogen-free conditions. Pmel-1 mice that carry T-cell receptor (TCR) transgene specific for the mouse homologue (pmel-17) of human glycoprotein gp100 were purchased from Jackson Laboratory (Bar Harbor, ME, USA). RENCA, a murine RCC line, stably transduced to express human CA9 (RENCA-CA9) was a gift from Dr Arie Belldegrun (University of California, Los Angeles). Human gp100-transduced B16 (B16-gp100), a murine melanoma line, was kindly provided by Dr Alexander Rakhmilevich (University of Wisconsin). These cells were maintained in Dulbecco's modified Eagle's medium and RPMI 1640, supplemented with 10% heat-inactivated fetal bovine serum (FBS; Life Technologies, Grand Island, NY, USA), 2 mmol l^−1^ of L-glutamine, 100 U ml^−1^ of penicillin, and 100 *μ*g ml^−1^ of streptomycin. The *in vitro* tumour cell growth studies are described in the supplemental methods. All animal studies were reviewed and approved by the Institutional Animal Care and Use Committee.

### Antibodies and reagents

Mouse monoclonal antibodies (mAbs) were purchased and used to bind CD8-*α* (53–6.7 PE-Cy5.5 conjugated, Biolegend, San Diego, CA, USA); Thy1.1 (OX-7, FITC conjugated, Biolegend); FoxP3 (150D, eBioscience, San Diego, CA, USA); CD62L (MEL-14, Biolegend); interferon (IFN)-*γ* (FITC conjugated, BD Biosciences Pharmingen, San Jose, CA, USA); DC marker CD11c (HL3, PE conjugated, BD Biosciences Pharmingen); MHC class I molecule H-2K^b^ (AF6-88.5, PE conjugated, BD Biosciences Pharmingen); MHC class II molecule I-A/I-E (2G9, FITC conjugated, BD Biosciences Pharmingen); co-stimulatory molecules CD80 (16-10A1, PE conjugated, BD Biosciences Pharmingen); and CD86 (GL1, PE conjugated, BD Biosciences Pharmingen). Immunostaining is described in supplemental material. Recombinant human interleukin (IL)-2 was purchased from Novartis Pharmaceuticals (Emeryville, CA, USA). The cDNA for mouse hsp110, human CA9 (a gift from Dr Arie Belldegrun), and human gp100 (a gift from Dr Nicholas Restifo, National Cancer Institute) were cloned into pBacPAK-his vector (BD Biosciences Clontech, Mountain View, CA, USA), and recombinant proteins were produced using the BacPAK baculovirus system according to the manufacturer's recommendations. CellTrace 5-(and 6-)carboxyfluorescein diacetate succinimidyl ester (CFSE) cell proliferation kit was purchased from Molecular Probes (Eugene, OR, USA). Temsirolimus and rapamycin were purchased from LC Laboratories (Woburn, MA, USA).

### Anti-tumour studies in mice

The HSP-based anti-tumour vaccines were generated by incubating and non-covalently complexing recombinant proteins; hsp110 was combined with gp100 or CA9 at an equal molar ratio as previously described (Wang *et al*, 2003, 2008). In the tumour treatment study, BALB/C mice were injected intradermally (i.d.) with vaccine consisting of 2 × 10^5^ RENCA-CA9 cells on day 0. C57BL/6 mice were injected i.d. with vaccine consisting of 2 × 10^5^ B16-gp100 cells on day 0. Starting day 10, mice were treated based on group assignment (five mice per group): group 1: control mice were treated with PBS (subcutaneously, s.c.) on days 10 and 17. Group 2: vaccine (25 *μ*g gp100 or CA9 complexed with an equal molar ratio of hsp110) was injected i.d. on days 10 and 17. Group 3: temsirolimus (15 *μ*g) was injected intraperitoneally (i.p.) on days 11–24. Group 4: group receiving vaccine plus temsirolimus was treated as described for groups 2 and 3. Tumours were measured every 3 days using an electronic caliper, and tumour volume was calculated as (shortest diameter^2^ × longest diameter/2). The tumour prevention study was performed similarly, except a single vaccine dose (day 0) was administered, temsirolimus was injected daily on days 8–32, and tumour cells were implanted on day 150.

### *In vitro* T-cell proliferation

For the [^3^H] thymidine incorporation assay, lymph nodes were harvested from naive C57 BL/6 or Pmel-1 mouse. In all, 3 × 10^5^ cells per well were cultured in 96-well plates and stimulated, with or without mTOR inhibitors, for 72 h. C57 BL/6 lymphocytes were stimulated with anti-CD3 and anti-CD28 mAb, and Pmel-1 lymphocytes were stimulated with gp100 peptide. DNA synthesis was determined by incubation for 16 h with 1 *μ*Ci [^3^H]thymidine (Amersham Biosciences, Piscataway, NJ, USA).

For the CFSE dilution assay, lymphocytes were harvested and treated as described above. The cells were labelled with 5 *μ*M CFSE, incubated at 37°C for 20 min, washed, and re-suspended in complete culture medium (RPMI 1640, 10% fetal calf serum, 2 mmol l^−1^ L-glutamine, 100 U ml^−1^ penicillin/streptomycin). Lymphocyte proliferation was assessed by flow cytometric analysis of CFSE dilution while gating on CD4 or CD8. To study lymphocyte proliferation in response to DC stimulation, bone marrow (BM) DCs were pulsed with antigens for 2 h, washed, treated with mTOR inhibitors for 2 h, and then washed again. Lymphocytes were harvested from Pmel-1 mice. CD8 T cells were purified by negative selection using mouse CD8 cell recovery column kit (Cedarlane, Ontario, Canada). Antigen-pulsed DC and CFSE-labelled lymphocytes were mixed at 1 : 10 ratio, and cultured for 48–72 h. Lymphocyte proliferation was assessed by flow cytometric analysis of CFSE dilution.

### Assays for T-cell function

The assays for T-cell function have been described previously (Wang *et al*, 2008). The ELISPOT assay, the *in vivo* CTL assay, and the intracellular IFN-*γ* staining are briefly described in the supplemental material.

### Adoptive transfers and treatment

To study T-cell memory, 3 × 10^4^ CD8^+^/Thy1.1^+^ lymphocytes from naïve Pmel-1 mice were adoptively transferred intravenously to C57BL/6 mice on day −1. On day 0, mice were immunised (complex of hsp110 and gp100) i.d., injected daily (i.p.) with temsirolimus (15 *μ*g ml^−1^ in 100 *μ*l PBS) from days 8 to 32, or treated with both. To assess the memory response, mice were re-challenged with vaccine on day 33. Spleen and lymph nodes were collected on days 18, 32, and 39. CD8, Thy1.1, and IFN-*γ*-positive cells were analysed by flow cytometry.

### Preparation of DCs from BM and characterisation of DC markers

The BMs were harvested from femurs and tibias, and treated with red cell lysis buffer, washed, and plated at a density of 1 × 10^6^ cells per ml in 12-well plates in RPMI 1640 containing 10% FBS and 10 ng ml^−1^ of recombinant mouse GM–CSF (eBioscience). Cells were fed every 2 days and harvested on days 7–9. In the culture, 75–90% of the cells were CD11c positive.

The BM DCs were stimulated for 24 h in complete medium with or without temsirolimus and washed 3 times with PBA (PBS with 0.1% bovine serum albumin (BSA) and 0.01% sodium azide). For FACS analysis, cells (3–5 × 10^5^) were suspended in 100 *μ*l PBS with 0.3% BSA and 0.05% sodium azide, and were stained with antibodies for 30 min on ice. After incubation, the cells were washed, and the fluorescence was measured by a FACScan (Becton, Franklin Lakes, NJ, USA). For each sample, fluorescence data from 10 000 cells were collected, and positive cells were expressed as the percent of total events.

## Results

### Effect of cancer vaccine and temsirolimus on tumour growth

**Figure 1:**
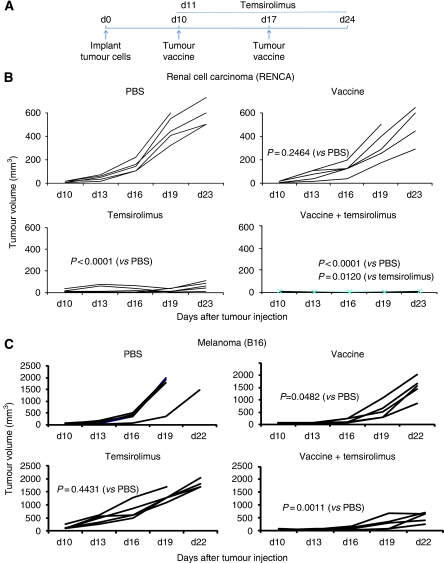
Combination therapy with HSP-based tumour vaccine and temsirolimus inhibits the growth of established tumours in models of renal cell carcinoma and melanoma. (**A**) A tumour treatment model was used to assess anti-tumour activity. After 10 days of s.c. implantation of syngeneic tumour lines, mice (five mice per group) were treated with PBS (control), tumour vaccine (days 10 and 17), temsirolimus (days 11–16, 18–23), or both, and tumour growth was monitored. (**B**) In a renal cell carcinoma model, RENCA-CA9 cells were implanted into BALB/C mice. The tumour vaccine was a non-covalent complex of recombinant hsp110 and CA9. Each line represents tumour growth in a single animal (**B** and **C**). *P*-values are provided comparing various groups using the repeated measures ANOVA. (**C**) In a melanoma model, B16-gp100 cells were implanted into B6 mice. The tumour vaccine was a non-covalent complex of recombinant hsp110 and gp100. Representative results are shown from at least two independent experiments.

The HSP-based vaccines consisting of a non-covalent complex of recombinant HSP and recombinant tumour antigen are effective for stimulating anti-tumour immunity in animal models (Wang *et al*, 2003; Kim *et al*, 2007). However, HSP-based vaccines have only a modest effect on growth of well-established murine tumours. The immune-modulating effects of temsirolimus were evaluated using an experimental model where vaccine therapy alone is insufficient. Before starting treatment, tumours that grow aggressively and require sacrifice of animals within ∼20 days were grown s.c. for 10 days until they were clearly palpable (Figure 1A). The average RENCA tumour was 49 mm^3^ and the average B16 tumour was 67 mm^3^ on day 10. In a RCC model, RENCA tumours expressing CA9 were treated with tumour vaccine (hsp110+CA9) and temsirolimus (Figure 1B). Temsirolimus alone produced some inhibition of tumour growth, indicating that temsirolimus has direct anti-tumour effect against RENCA. However, the combination of tumour vaccine and temsirolimus eradicated the tumour in all animals.

**Figure 2:**
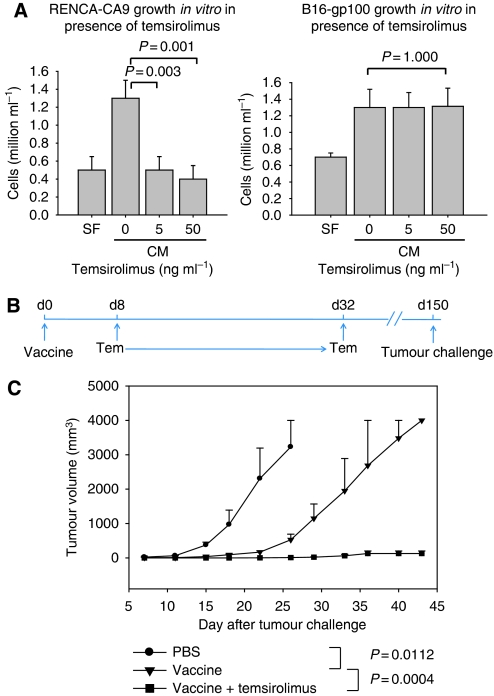
Temsirolimus can have a direct anti-proliferative effect on the tumour; however, temsirolimus can also prevent tumour growth by enhancing anti-tumour immunity. (**A**) Direct anti-tumour effects of temsirolimus were assessed for RENCA and B16 cell lines *in vitro.* Data show mean and s.e.m. values. Representative results are shown from at least three experimental repeats. (**B**) In a murine tumour prevention model, B6 mice (six mice per group) were treated with PBS (day 0), tumour vaccine (day 0), or tumour vaccine plus temsirolimus (days 8–32). (**C**) Mice were challenged with B16-gp100 cells, and tumour growth was monitored. The tumour vaccine was a non-covalent complex of recombinant hsp110 and gp100. Mean tumour growth and s.e.m. are provided, and *P*-values were determined using repeated measures ANOVA. CM=complete medium, SF=serum-free medium.

In a murine melanoma model, B16 melanoma cells expressing gp100 were implanted 10 days before starting treatment with tumour vaccine (hsp110+gp100) and temsirolimus (Figure 1C). The combination of vaccine and temsirolimus was more effective in inhibiting tumour growth than either therapy alone. The inhibition of tumour growth may have been mediated, in part, by direct cytotoxic effect as well as indirect anti-tumour immune effects. Therefore, to assess the direct anti-tumour effects of temsirolimus on RENCA and B16, both cell lines were cultured in the presence of varying concentrations of temsirolimus (Figure 2A). RENCA was sensitive to temsirolimus; however, B16 was unaffected by concentrations of temsirolimus several folds higher than expected in mouse serum (Guba *et al*, 2005), suggesting that the inhibition of these tumours *in vivo* is at least, in part, immune mediated.

To firmly establish a temsirolimus-mediated immune mechanism for inhibition of tumour growth, a tumour prevention model was used (Figure 2B). In the B16 melanoma model, mice (six per group) were treated with a single dose of tumour vaccine with or without temsirolimus, and challenged with B16 tumour cells after more than 100 days following the last temsirolimus administration. Therefore, temsirolimus could not have had any direct cytotoxic effects on the B16 tumours. Administration of vaccine alone had a significant inhibitory effect on the growth of B16 tumours (Figure 2C). However, the administration of both vaccine and temsirolimus prevented the growth of tumour in all mice, and animals remained tumour free during over 60 days of observation. Therefore, temsirolimus augments the anti-tumour immunity induced by HSP-based cancer vaccines.

As a class, mTOR inhibitors are well-characterised immune suppressors. Therefore, these observations are surprising. In the following experiments, the immunostimulatory and immunosuppressive properties of temsirolimus were characterised in our model.

### Immunosuppressive effects

#### Effect of temsirolimus on T-cell proliferation.

**Figure 3:**
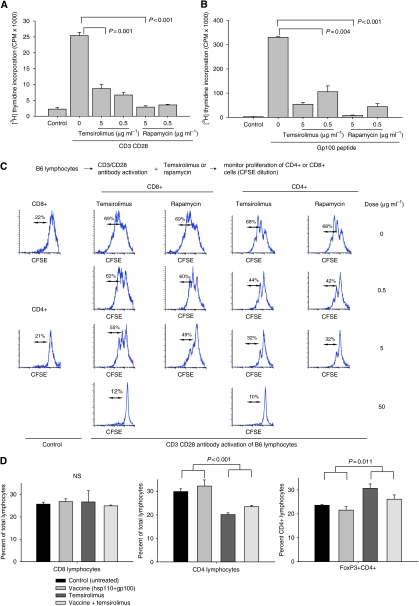
Temsirolimus decreases proliferation of activated T cells and increases the percent of CD4 cells that express FoxP3. (**A** and **B**) Proliferation of activated lymphocytes was assessed *in vitro* by monitoring [^3^H] thymidine incorporation. B6 lymphocytes were stimulated with anti-CD3 and anti-CD28 mAb (**A**), and Pmel-1 lymphocytes were stimulated with gp100 peptide (**B**). Lymphocytes were stimulated in the presence of varying concentrations of temsirolimus or rapamycin, and proliferation was monitored. (**C**) Proliferation of activated CD4 or CD8 T cells was assessed *in vitro* with CFSE dilution assays. B6 lymphocytes were labelled with CFSE and stimulated with anti-CD3 and anti-CD28 mAb in the presence of varying concentrations of temsirolimus or rapamycin. The CFSE profiles were monitored after gating on CD8 or CD4. (**D**) *In vivo* effects of temsirolimus on CD4 or CD8 T cells were assessed. B6 mice (five mice per group) were treated daily with temsirolimus for 24 days, two doses of vaccine (complex of hsp110 and gp100), or both. CD4 and CD8 lymphocytes were assessed by flow cytometry. FoxP3 staining was quantified while gating on CD4. Data show mean and s.e.m. values Representative results are shown from at least two independent experiments.

Temsirolimus is an analogue of the classic mTOR inhibitor, rapamycin. Both temsirolimus and rapamycin inhibited the proliferation of activated T cells *in vitro* (Figures 3A and B). The [^3^H] thymidine incorporation assay showed that mTOR inhibition decreased proliferation of bulk T cells. The CFSE dilution assays were then performed to assess the effects of mTOR inhibition on specific populations of T cells (Figure 3C). Temsirolimus and rapamycin inhibited the proliferation of activated CD8 T cells and CD4 T cells in a dose-dependent manner *in vitro*. However, the *in vivo* effects were less pronounced (Figure 3D). Following treatment of B6 mice with temsirolimus for 24 days, the percent of lymphocytes that were CD8+ was unchanged by temsirolimus. The percent of CD4 lymphocytes was decreased in all groups that received temsirolimus; however, the percent of CD4 cells that were also FoxP3+ was increased by temsirolimus. Therefore, temsirolimus increased the relative abundance of regulatory CD4 cells.

#### Effect of temsirolimus on DCs.

**Figure 4:**
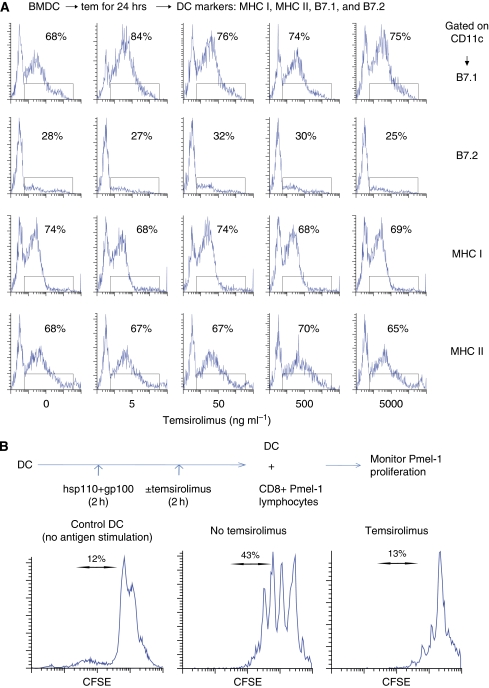
Treatment with temsirolimus has minimal effect on baseline expression of DC markers; however, temsirolimus decreases the capacity of DCs to stimulate T-cell proliferation. (**A**) To assess baseline DC markers, BM-derived DCs were treated *in vitro* for 24 h with varying doses of temsirolimus. The DCs were examined by flow cytometry while gating on CD11c. (**B**) To assess DC function, BM-derived DCs were stimulated with vaccine (complex of hsp110 and gp100) and then treated with temsirolimus (50 ng ml^−1^). These DCs were then used to stimulate CD8 T cells purified by negative selection from Pmel-1 lymphocytes. Proliferation of Pmel-1 T cells was assessed by monitoring CSFE dilution while gating on CD8. Representative results are shown from at least three independent experiments.

The mTOR pathway is involved in many key functions performed by DCs (Thomson *et al*, 2009). Others have documented that during LPS stimulation, mTOR inhibition can decrease the expression of DC markers that are critical to its function (Hackstein *et al*, 2002; Monti *et al*, 2003). In our study, baseline DC markers were essentially unaffected by varying concentrations of temsirolimus applied for 24 h (Figure 4A). However, when generating anti-tumour immunity, the ability to stimulate T cells is ultimately the most critical DC function. Therefore, DCs were stimulated with hsp110+gp100 and then treated with temsirolimus (Figure 4B). These DCs were then used to stimulate CD8 T cells purified from Pmel-1 transgenic mice, which carry a rearranged TCR that recognises a gp100 epitope (amino acids 25–33) presented by H2-D^b^ MHC class I molecules. Temsirolimus treatment decreased the ability of DCs to stimulate Pmel-1 CD8 T-cell proliferation.

### Immunostimulatory effects

#### Activation of CD8 T cells.

**Figure 5:**
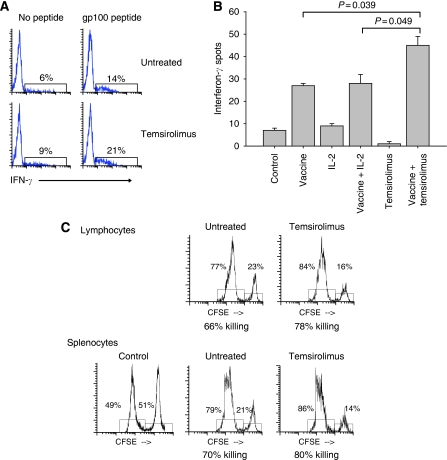
Temsirolimus enhances the activation of T cells. (**A**) The stimulation of antigen-specific IFN-*γ* response from CD8 T cells was examined *in vitro*. Pmel-1 splenocytes were harvested, stimulated with gp100 peptide, and examined for IFN-*γ* staining by flow cytometry while gating on CD8. (**B**) The *in vivo* gp100-specific immune response was measured using an ELISPOT assay. B6 mice were treated with vaccine (complex of hsp110 and gp100), temsirolimus (daily for 14 days), or both. Treatment with IL-2 (5 × 10^4^ U per mouse daily for 14 days) was included for comparison. (**C**) T-cell activation and killing ability were assessed using an *in vivo* cytotoxic T-lymphocyte assay. Pmel-1 lymphocytes were adoptively transferred into B6 mice. The mice were treated once with cancer vaccine, with or without 7 days of temsirolimus. Splenocytes from naive mice pulsed with murine gp100 peptide were fluorescently labelled and used as target cells. Splenocytes and lymphocytes were harvested after 14 h and analysed by flow cytometry. Representative results are shown from at least two independent experiments. IL-2=interleukin-2.

The effect of temsirolimus on T-cell activation was evaluated both *in vitro* and *in vivo.* Splenocytes from Pmel-1 mice were harvested and treated with gp100 peptide with or without temsirolimus (Figure 5A). Splenocytes expressing CD8 and staining for IFN-*γ* were increased in the group treated with temsirolimus. The IFN-*γ* response was also measured with an ELISPOT assay (Figure 5B); treatment of B6 mice with hsp110+gp100 produced a gp100-specific IFN-*γ* response that was significantly increased by temsirolimus. Interleukin-2 is an immunostimulatory cytokine that can enhance a cellular immune response and was included for comparison. The combination of vaccine and temsirolimus produced a greater IFN-*γ* response than the combination of vaccine and IL-2. Finally, an *in vivo* CTL assay was performed (Figure 5C). Temsirolimus increased the killing of gp100 peptide-treated target cells. These results show that temsirolimus enhances the activation and function of effector T cells stimulated with an HSP-based anti-tumour vaccine.

#### Memory T-cell response.

**Figure 6:**
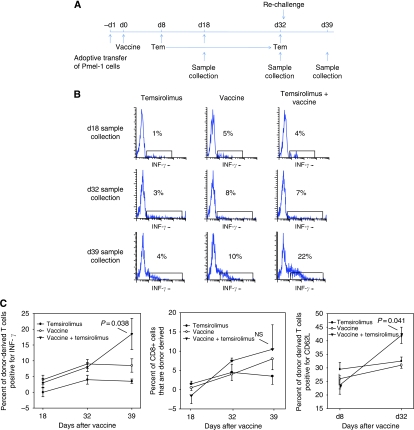
Temsirolimus enhances memory T-cell response by producing centrally homing memory T cells capable of enhanced IFN-*γ* response on re-challenge with tumour antigen. (**A**) To monitor memory T-cell formation and function, Pmel-1 lymphocytes were adoptively transferred into B6 mice (five mice per group) on day −1. On day 0, mice were treated with vaccine (complex of hsp110 and gp100). Temsirolimus was administered during the T-cell contraction phase (days 8–32). Mice were re-challenged with vaccine on day 33. (**B**) Lymphocytes were harvested on days 18, 32, and 39, and lymphocytes were assessed for IFN-*γ* while gating on Thy1.1, which marks donor-derived lymphocytes. A representative flow cytometry result is shown. (**C**) From the same experiment, the mean and s.e.m. values for the donor-derived IFN-*γ* response are provided (left panel). To assess proliferation of donor-derived CD8 memory T cells, the percent of CD8 T cells expressing Thy1.1 was assessed before and after re-challenge with antigen (middle panel). CD62L expression was measured in donor-derived memory lymphocytes before and after starting temsirolimus (days 8 and 32; right panel). Representative results are shown from two independent experiments.

Memory T cells are critical for generating effective anti-tumour immunity. The tumour prevention study presented in Figure 2B and C suggests that the combination of cancer vaccine and temsirolimus is highly effective in generating immune memory. To characterise the memory T cells formed by the combination therapy, Pmel-1 lymphocytes were adoptively transferred into B6 mice (Figure 6A). The donor lymphocytes were stimulated by treating the B6 mice with hsp110+gp100 (day 0). Temsirolimus was administered during the transition from effector phase to memory phase (days 8–32). Donor-derived lymphocytes were monitored by flow cytometry while gating on Thy1.1; by day 32, all surviving donor-derived lymphocytes were considered memory cells.

To assess the ability of memory cells to re-activate, percent of donor-derived lymphocytes that stain for IFN-*γ* was assessed (Figure 6B). Temsirolimus had no effect on percent of donor cells staining for IFN-*γ* in samples collected before repeat challenge with vaccine (i.e., days 18 and 32). However, following re-stimulation on day 33, the percent of donor cells collected on day 39 that stained for IFN-*γ* was highest in the group treated with both vaccine and temsirolimus (Figure 6C). Interestingly, proliferation of CD8 T cell following re-stimulation was not significantly different between any of the treatment groups and the control group, suggesting that temsirolimus enhances memory CD8 T-cell function rather than proliferation. Consistent with this possibility, a higher percent of the memory cells stimulated by the cancer vaccine in the presence of temsirolimus was positive for CD62L, which is a marker for the more effective central memory cells.

## Discussion

The mTOR protein has a critical role in integrating diverse environmental signals that ultimately affect cell growth and proliferation. Inhibition of mTOR function has a large number of cellular effects that include inhibition of proliferation. Temsirolimus is a rapamycin analogue and a potent mTOR inhibitor that is FDA approved for the treatment of advanced RCC. We characterised effects of temsirolimus when used in combination with a novel cancer vaccine. It is surprising that temsirolimus can enhance anti-tumour immunity, as rapamycin is widely used in the clinic to suppress the immune system following solid-organ transplantation. In an experimental murine model of RCC (RENCA), the combination of an HSP-based cancer vaccine and temsirolimus was more effective against established tumours than either agent alone. This observation was confirmed in an experimental murine model of melanoma (B16), which is another classic immunoresponsive malignancy. In both models, untreated animals were needed to be killed ∼20 days following tumour implantation. To make the study rigorous, tumours were allowed to grow for 10 days until they were palpable and had an established vascular supply before starting treatment.

Temsirolimus had direct anti-proliferative effects on RENCA cells *in vitro*. However, there was no direct effect on the growth of B16 cells, suggesting that immune modulation may have produced the anti-tumour effects seen in the animal studies. To provide definitive evidence that mTOR inhibition can positively modulate an immune response, a tumour prevention model was used to isolate the immune effects of combination therapy. Mice were challenged with tumour cells for more than 100 days following treatment with cancer vaccine and temsirolimus. As the tumour cells were never directly exposed to temsirolimus, all anti-tumour effects could be attributed to immunostimulation. The combination therapy completely inhibited tumour growth, whereas treatment with cancer vaccine alone did not. Therefore, temsirolimus enhanced anti-tumour immunity.

The anti-tumour studies demonstrated that the net effect of immune modulation with mTOR inhibition was to enhance the cancer vaccine. However, mTOR inhibitors are known to have both immune-stimulating and immune-suppressing effects. To better understand these opposing functions, the immune effects of mTOR inhibition were characterised in our model. A detailed understanding of immune modulation may shed light on the mechanism of action of pharmacological mTOR inhibition, and identify strategies to enhance a particularly mTOR function and achieve varied clinical objectives.

Temsirolimus and rapamycin inhibited the proliferation of both activated CD8 and CD4 T cells *in vitro*. This observation is consistent with previous reports that rapamycin directly inhibits T-cell activation and proliferation (Mondino and Mueller, 2007; Song *et al*, 2007). In our study, the *in vivo* effects of mTOR inhibition differed for CD8 and CD4 T cells. When temsirolimus was administered to mice, the percent of CD8 lymphocytes in lymph nodes did not change, whereas the percent of CD4 lymphocytes decreased. CD4 lymphocytes include both effector and regulatory lymphocytes. Despite an overall decrease in CD4 lymphocytes, the percent of CD4 lymphocytes expressing FoxP3 increased in groups that received temsirolimus. This observation is consistent with reports that regulatory T cells are less sensitive to the anti-proliferative effects of mTOR inhibition (Battaglia *et al*, 2006; Segundo *et al*, 2006). In transplant patients treated with mTOR inhibition, an increase in regulatory T cells is believed to be an important mechanism for immune suppression. Therefore, the effects of mTOR inhibition on lymphocyte proliferation and regulatory T-cells are expected to favour immune suppression.

However, effector T cells generated *in vivo* during treatment with mTOR inhibitor had greater activity as measured using an ELISPOT assay. In a confirmatory study, the increased IFN-*γ* response was localised to CD8 T-cells. Furthermore, temsirolimus enhanced *in vivo* killing by cytotoxic T lymphocytes. These results suggest that the primary immune response generated by the cancer vaccine was enhanced by temsirolimus. It is possible that the regulatory T cells that increase during temsirolimus treatment ultimately suppress the CD8 T cell response; however, another possibility is that the increase in regulatory T cells is a negative feedback response to the enhanced cytotoxic T-cell function.

Previous reports do not produce a clear picture of the effects of mTOR inhibition on DC function. For example, rapamycin has been shown to suppress DC maturation by downregulating the IL-4 receptor complex and decreasing the production of IL-2 and tumour necrosis factor (Hackstein *et al*, 2003). Also, human DCs matured from monocytes in the presence of rapamycin had decreased ability to take up antigen, including decreased endocytosis and phagocytosis (Monti *et al*, 2003). However, other recent reports suggest that mTOR inhibition may enhance the innate immune response in DCs (Weichhart *et al*, 2008) and stimulate autophagy, which can enhance antigen presentation (Jagannath *et al*, 2009). We found no evidence that temsirolimus promotes anti-tumour immunity through its effects on DCs. Temsirolimus did not change the expression of baseline DC markers. In fact, BM-derived DCs stimulated with cancer vaccine and temsirolimus had decreased ability to stimulate CD8 T cell when compared with DCs stimulated with vaccine alone. The mTOR inhibition had a suppressive effect on overall DC function. Therefore, the effects of mTOR inhibition on DCs do not explain the enhanced anti-tumour immunity seen in our models.

We also characterised the effects of temsirolimus on CD8 memory cell formation. To minimise the effects of temsirolimus during the T-cell expansion phase, temsirolimus was started during T-cell contraction. This treatment schedule focuses the effects of temsirolimus on the transition from effector CD8 T cells to memory cells. Mice were re-stimulated with tumour antigen 33 days following primary vaccination when all transferred T cells should be long-lived memory cells. Samples collected 7 days after the re-stimulation show that CD8 T cells from animals treated with both vaccine and temsirolimus have an enhanced IFN-*γ* response when compared with controls; however, CD8 T-cell proliferation was not significantly enhanced by temsirolimus. This is consistent with a recent report that rapamycin treatment during T-cell contraction does not alter the number of CD8 T cells, but rather accelerates memory differentiation and produces T cells with phenotypic characteristics of highly functioning memory cells (Araki *et al*, 2009). We found that combined treatment with cancer vaccine and temsirolimus produced memory cells with greater CD62L expression than control treatments, indicating that temsirolimus promotes formation of central memory, which is associated with enhanced anti-tumour activity (Klebanoff *et al*, 2005).

The RCC and melanoma are classic immunoresponsive tumours. Novel cancer vaccines are being actively developed for these and other malignancies. Our results provide a rationale for combining mTOR inhibitors with novel and established immune therapies. Temsirolimus is currently approved for the treatment of RCC, and is therefore particularly attractive for use with cancer vaccines that are being investigated for RCC. Future studies will need to develop optimal dosing strategies and identify cancer vaccines that are best suited for use with mTOR inhibitors.

### Summary

Temsirolimus is an anti-neoplastic agent that is currently approved for patient use. It has immune-modulating activity. In animal models for studying tumour vaccines, temsirolimus enhanced vaccine activity by enhancing effector T-cell function and enhancing the production of CD8 memory T cells.
