# Supplementary material for: Temsirolimus, an mTOR inhibitor, enhances anti-tumour effects of heat shock protein cancer vaccines
Source: Br J Cancer. 2011 Feb 1;104(4):643–52. doi: 10.1038/bjc.2011.15 (PMC3049595; doi:10.1038/bjc.2011.15)
Supplement: Supplementary Information [file bjc201115x1.doc]

SUPPLEMENT

**Additional Materials and Methods**

Briefly, the **ELISPOT assay** was performed by harvesting lymph nodes (LN) and spleen 2 wks after immunization. Filtration plates (Millipore, Bedford, MA) were coated with 10 μg/ml rat antimouse INF-γ (clone R4-6A2; PharMingen, San Diego, CA) at 4°C overnight, washed and blocked. Lymphocytes (5 × 10^5^/well) were added and incubated with CA9 (10 μg/ml) at 37°C for 48 h, then washed. Biotinylated IFN-γ antibody (5 μg/ml; clone XMG1.2; PharMingen), avidin-alkaline phosphatase D (0.2 unit/ml; Vector Labs, Burlingame, CA) and 5-bromo-4-chloro-3-indolyl phosphate/nitroblue tetrazolium (Boehringer Mannheim, Indianapolis, IN) were used to detect IFN-γ secretion. IFN-γ spots were counted using the KS Elispot System (version 4.3.56) from Zeiss Microscopy (Oberkochen, Germany).

Briefly, the ***in vivo* CTL assay** was performed using single-cell suspensions of splenocytes (1 x 10^7^ cells/ml) from naïve mice with or without 10 µM peptide in DMEM containing 10% FBS for 30 min at 37°C. Each cell population was then labeled with a different concentration of CFSE (0.5 or 12.5 µM) at 2 x 10^7^cells/ml in PBS/0.1%BSA. CFSE labeling was stopped by addition of an equal volume of FBS for 1 min, and washed with RPMI complete medium, 3 times. 5 x 10^6^ cells of each peptide-pulsed or unpulsed population were mixed together and injected i.v. into immunized and unimmunized mice. Sixteen hours following transfer, mice were sacrificed, and splenocytes were harvested. Single-cell suspensions of splenocytes were prepared, and analyzed by flow cytometry. Percent-specific-lysis of fluorescent donor splenocytes was calculated as follows: [(number of unpulsed targets x A - number of pulsed targets)/number of unpulsed targets x *A*] x 100, where *A* = [number of pulsed targets / number of unpulsed targets] in unimmunized recipient mice.

For **intracellular IFN-γ staining**, lymphocytes recovered from culture were restimulated with PMA (20ng/ml) and ionomycin (400ng/ml) for 4 hrs in presence of 10 μg/ml brefeldin A. Lymphocytes were stained for cell surface antigens (e.g. CD8 or Vα-1), fixed, permeabilized, and stained for intracytoplasmic antigens (e.g. IFN-γ). Flow cytometry was performed using the FACScan (Becton-Dickinson, La Jolla, CA) and typically 10,000 live CD8^+^ and/or CD4^+^ gated events were analyzed using Winlist software(Verity, Topsham, ME).

For the ***in vitro* analysis of tumor cell growth**, cells were seeded at 10% confluence (~1–5 × 10^4^ tumor cells) into 24-well culture plates using the appropriate complete growth medium. On day 3, Replicate cultures were treated with serum free medium, complete growth medium, or complete growth medium containing serially diluted temsirolimus (3 wells for each treatment). On day 4, the cells were released by trypsin digestion and the number of viable (trypan blue dye-excluding) cells counted using a hemocytometer.
